# Zonation of Nitrogen and Glucose Metabolism Gene Expression upon Acute Liver Damage in Mouse

**DOI:** 10.1371/journal.pone.0078262

**Published:** 2013-10-17

**Authors:** Shahrouz Ghafoory, Katja Breitkopf-Heinlein, Qi Li, Catharina Scholl, Steven Dooley, Stefan Wölfl

**Affiliations:** 1 Institute of Pharmacy and Molecular Biotechnology, Heidelberg University, Heidelberg, Germany; 2 Department of Medicine II, Section Molecular Hepatology - Alcohol Associated Diseases, Medical Faculty Mannheim, Heidelberg University, Mannheim, Germany; Massachusetts Institute of Technology, United States of America

## Abstract

Zonation of metabolic activities within specific structures and cell types is a phenomenon of liver organization and ensures complementarity of variant liver functions like protein production, glucose homeostasis and detoxification. To analyze damage and regeneration of liver tissue in response to a toxic agent, expression of liver specific enzymes was analyzed by *in situ* hybridization in mouse over a 6 days time course following carbon tetrachloride (CCl_4_) injection. CCl_4_ mixed with mineral oil was administered to BALB/c mice by intraperitoneal injection, and mice were sacrificed at different time points post injection. Changes in the expression of albumin (Alb), arginase (Arg1), glutaminase 2 (Gls2), Glutamine synthetase (Gs), glucose-6-phosphatase (G6pc), glycogen synthase 2 (Gys2), Glycerinaldehyd-3-phosphat-Dehydrogenase (Gapdh), Cytochrom p450 2E1 (Cyp2e1) and glucagon receptor (Gcgr) genes in the liver were studied by *in situ* hybridization and qPCR. We observed significant changes in gene expression of enzymes involved in nitrogen and glucose metabolism and their local distribution following CCl_4_ injury. We also found that Cyp2e1, the primary metabolizing enzyme for CCl_4_, was strongly expressed in the pericentral zone during recovery. Furthermore, cells in the damaged area displayed distinct gene expression profiles during the analyzed time course and showed complete recovery with strong albumin production 6 days after CCl_4_ injection. Our results indicate that despite severe damage, liver cells in the damaged area do not simply die but instead display locally adjusted gene expression supporting damage response and recovery.

## Introduction

Liver is the central metabolic organ in vertebrates and plays key roles in many physiological processes, including detoxification, synthesis of plasma proteins, glucose homeostasis, as well as utilization and cycling of various nutrients. Loss of liver function is the consequence of various liver diseases and toxic damage, and is a major health risk factor. The liver is also known for its high capacity for tissue regeneration. In response to damage, tissue repair mechanisms are initiated, enabling regeneration of the damaged tissue [1]. In certain conditions, e.g. severe damage, viral infections and continuous exposure to toxic chemicals, dysfunctional tissue repair can also lead to degenerative liver disease, including liver fibrosis, cirrhosis and hepatocellular carcinoma (HCC). In this study we used the well-known hepatotoxin carbon tetrachloride (CCl_4_) to induce tissue damage [2, 3] and followed the regeneration of the tissue in a 6 days time course analyzing the expression of key enzymes of major metabolic pathways by *in situ* hybridization (ISH) to elucidate the interplay between damage response and maintenance of liver function in the functional units of the liver. 

The liver is organized in basic functional units called acini. These units consist of two regions, an upstream area around the terminal portal vein and the terminal hepatic arteriole (periportal) and a downstream area around the central vein (pericentral). These two zones are unequally involved in metabolic processes reflected in distinct expression patterns of enzymes. The periportal area has a greater ability for glucose output, urea synthesis and bile formation, whereas glucose uptake, glutamine synthesis and xenobiotic metabolism dominantly takes place in the pericentral area [4, 5, 6]. 

### Nitrogen metabolism

**Figure 1:**
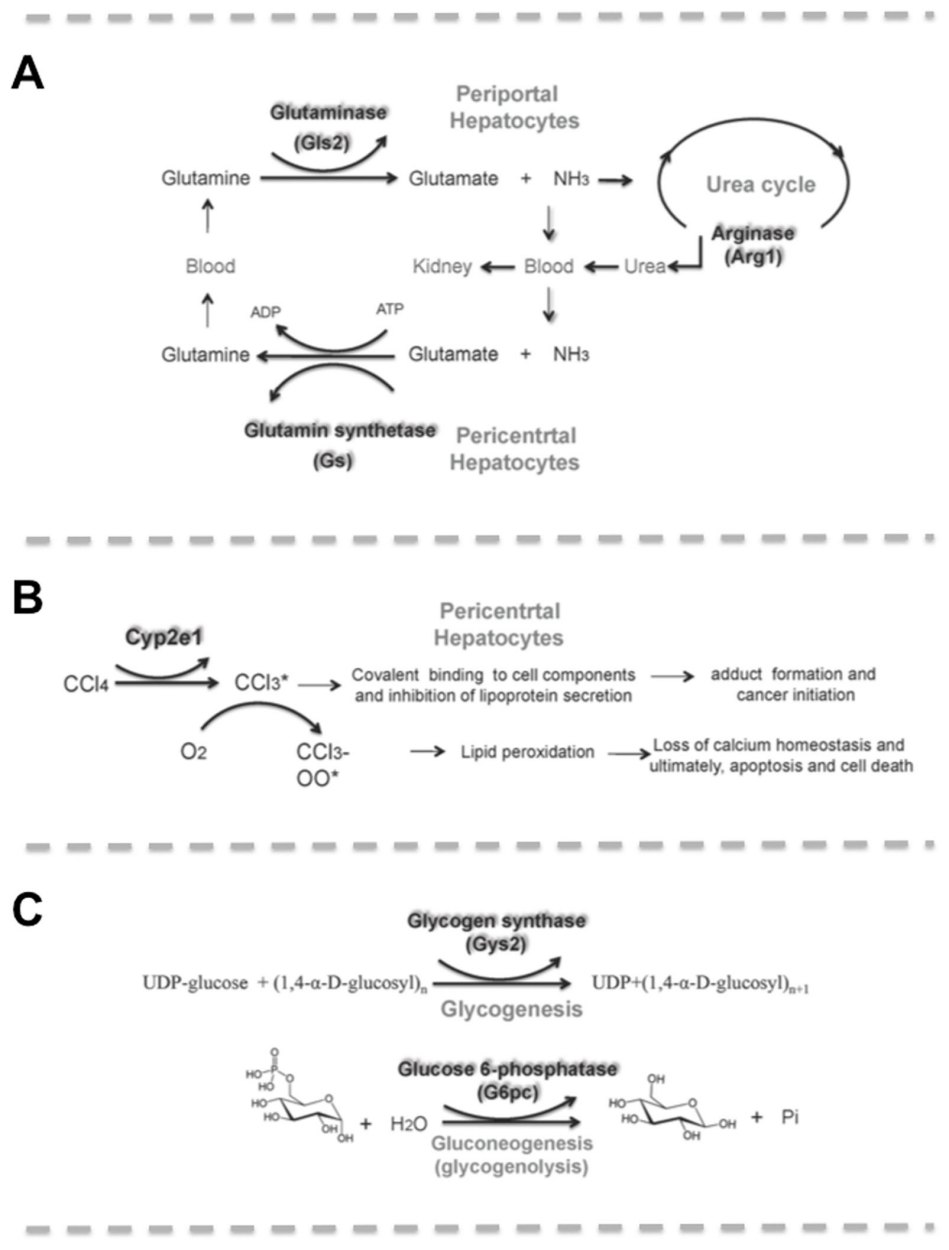
Scheme of enzymatic reactions involved in basic liver functions and metabolism. (**A**) Function of key enzymes of nitrogen metabolism and their zonation in healthy liver hepatocytes. (**B**) Enzymatic reactions involved in glucose storage and release in hepatocytes. (**C**) Metabolic activation of CCl_4_ in pericentral hepatocytes.

Urea and glutamine synthesis in the liver play a central role in ammonia detoxification [7] (Figure 1A). These two major ammonia detoxification reactions are separated within the liver acini, where they are anatomically arranged in series, with urea synthesis in the periportal area and glutamine synthesis and absorption of ammonia mainly in the perivenous area. Thus, ammonia escaping from the periportal urea synthesis is scavenged in the perivenous area [8].

Most nitrogen from peripheral tissue is not transported as free ammonia but as amino acids, such as glutamine, which is absorbed by periportal hepatocytes (PPH). There it is hydrolyzed by glutaminase 2 (Gls2) into glutamate and ammonia, the latter further directed to the urea cycle and detoxified through conversion into urea [9]. In the last step of the urea cycle arginine is converted into urea and ornithine by arginase 1 (Arg1) [9, 10]. In the pericentral area glutamate and ammonia are absorbed from blood by 2-3 hepatocyte layers around the central vein and used to produce glutamine by glutamine synthetase (Gs) [4, 10] (Figure 1A). 

### Carbohydrate metabolism

In the control of glucose homeostasis, liver can store surplus carbohydrates in form of glycogen, which can be rapidly mobilized when needed to maintain blood glucose levels [11]. In hepatocytes glycogen is synthesized by glycogen synthase 2 (Gys2) from glucose-6-phosphate (Figure 1B), which is either derived from glucose absorbed from blood or from gluconeogenesis utilizing other precursors, like pyruvate, lactate or glutamine. Utilization of glucose from blood occurs mainly in pericentral hepatocytes (PCH), while gluconeogenesis is dominant in periportal hepatocytes (PPHs). Thus, the two glycogen synthesis routes are taking place within different metabolic zones of the liver [12]. A link is the production of lactate by glycolysis, which is released by PCHs and at least in part reabsorbed and used for gluconeogenesis by PPHs. [5, 12, 13, 14].

Glucose-6-phospate is the central metabolite of glucose metabolism and provides the connection between glycolysis, gluconeogenesis, glycogen synthesis and glycogenolysis [15]. For release of glucose, Glucose-6-phospate is hydrolyzed by glucose-6-phosphatase (G6pc). In the liver, glucose-6-phosphatase activity is higher in PPHs than in PCHs as shown by a quantitative histochemical study [16]. In all hepatocytes glucose is utilized for energy production by glycolytic degradation, which involves the catalytic conversion of glyceraldehyde-3-phosphate to 1,3-biphosphoglycerate through glyceraldehyde 3-phosphate dehydrogenase (Gapdh) [17]. 

### Liver damage

Liver damage upon CCl_4_ treatment depends on dose and duration of exposure. A single dose of CCl_4_ can induce transient damage, while extended exposure, with repeated administration of CCl_4_, leads to consistent tissue damage and liver degeneration, including fibrotic transformation, cirrhosis and hepatic cancer formation [2, 3, 18]. In hepatocytes CCl_4_ is activated by cytochrome p450 2E1 (Cyp2e1) to form the trichloromethyl radical, CCl_3_* [2](Figure 1C). This radical can react with various cellular molecules (e.g. nucleic acid, protein, lipid), impairing cellular processes. In presence of excess oxygen, CCl_3_* will react with oxygen to form the trichloromethylperoxy radical CCl_3_OO*, another highly reactive species. This molecule can cause lipid peroxidation, and destroys polyunsaturated fatty acids, which contributes to severe cell damage[2, 3]. 

Cyp2e1 is predominantly expressed in PCHs. Consequently, treatment with CCl_4_ leads to damage of PCHs, while PPHs are not damaged [19,20]. The damage of PCHs results in reduced glutamine synthase activity, impairing glutamine metabolism and ammonia detoxification, although periportal urea synthesis is not changed[21].

CCl_4_ treatment also influences carbohydrate metabolism. In perfusion experiments with CCl_4_ treated livers specifically glycogen synthesis from exogenous glucose, but not from gluconeogenesis, was disturbed [22]. Treatment with CCl_4_ also reduced glucose-6-phosphatase activity of isolated liver microsomes [23].

Here we present the expression of key metabolizing enzymes in different zones of liver acini at single cell resolution following a single CCl_4_ treatment over 6 days using *in situ* hybridization. Our analysis revealed distinct zones of gene expression for most genes analyzed confirming a clear spatial distribution of metabolic activity. Cells in the damaged area were able to adjust gene expression, clearly indicating that hepatocytes remained viable in the “damaged” pericentral area. 

## Materials and Methods

### Animal model and carbon tetrachloride (CCl_4_) treatment

In this study, 28 (8-week-old) Balb/c mice weighing 20–25g were used. Acute liver injury was induced by intraperitoneal injection of CCl_4_ mixed with mineral oil (1ml ⁄kg body weight). All animals received humane care and all animal protocols were in full compliance with the guidelines for animal care and were approved by the government of Baden-Württemberg’s Animal Care Committee, Regierungspräsidium Karlsruhe, Germany.

 Mice were killed at different time points after CCl_4_ injection (3h, 6h, and on days 1, 2, 3 and 6 post-injection). Liver pieces were rinsed in RNase-free phosphate-buffered saline (PBS) and fixed immediately with 4% paraformaldehyde (PFA) at 4°C overnight (O/N). Fixed tissue was washed with PBS, dehydrated with increasing ethanol concentrations (35- 96%) and subsequently embedded in paraffin.

### Preparation of tissue sections

Paraffin sections were cut into 4μm slices, mounted on poly-l-lysine-coated slides, air-dried O/N at 37°C and stored at 4°C (48h). For ISH paraffin sections were dewaxed and rehydrated, treated with proteinase K, washed with glycin (0.2%) and fixed with 4% PFA.

### *In situ* hybridizaion

Gene specifc template PCR-fragments were generated from cDNA synthesized from total RNA of C57BL/6 mouse liver as described previously [24] using primers for selected genes listed in Table 1. Hybridization probes were prepared from template PCR-fragments with Sp6 and T7 promoters for the synthesis of sense (SP6 RNA polymerase) and anti-sense (T7 RNA polymerase) cRNA probes as described [24] using digoxigenin and fluorescein-labeled nucleotide mix (Roche, Mannheim, Germany) and SP6 or T7 RNA polymerase (Fermentas). Labeled cRNA probes were dissolved in 50% Formamide/2x SSC standard saline citrate (SSC) and *in situ* hybridization was performed as described [24]. For double staining hybridization was performed simultaneously with one digoxigenin and one fluorescein labeled probe [24]. Images were taken using a digital microscope (Biorevo 9000, Keyence, Japan) with two different magnifications (4x or 20x objective).

**Table 1:** Primers used for preparation of *in situ* hybridization probes.

Gene name	NCBI Reference Sequence	Forward	Reverse
Albumin	NM_009654.3	CCTGCAACACAAAGATGACAACCCC	GGGATCCACTACAGCACTTGGTAAC
Arginase (Arg1)	NM_007482.3	GAGCTCCAAGCCAAAGTCCTTAGAG	CGAAGCAAGCCAAGGTTAAAGCCAC
Glutaminase 2 (Gls2)	NM_001033264.3	CTTAGGCACTGACTACGTGCACAAG	CCGAGACATCTCCACTATATGCAGC
Glutamine synthetase	NM_008131.3	CTCCATCCTGTTGCCATGTTTCGAG	GAGAGGGATCACTGGAAGTCTAGTC
Glucose-6-phosphatase (G6pc)	NM_008061.3	CCCATCCCAGGTTGAGTTGATCTTC	GAGAGAAGAATCCTGGGTCTCCTTG
Glycogen synthase 2 (Gys2)	NM_145572.2	CTGGGTTCATGTGACCTCAGATTGC	CCTCGATGGCTGTGATTTCTGACAC
Gapdh	NM_008084.2	GAGTATGTCGTGGAGTCTACTGGTG	GGTTTCTTACTCCTTGGAGGCCATG
Cytochrome P450 (Cyp2e1)	NM_021282.2	CAAGGAGGTGCTACTGAACCACAAG	GATGACATATCCTCGGAACACGGTG
Glucagon receptor (Gcgr)	NM_008101.2	CACAGTGATCATGCAGTACGGCATC	GTGCACAGTACAAGCTGCTGTCTTG
Alpha-smooth muscle actin (aSma)	NM_007392.2	GAAGAGCATCCGACACTGCTGACAG	CAGTTGTGTGCTAGAGGCAGAGCAG

### Immunohistochemistry

Immunohistochemistry for alpha-smooth muscle actin (aSma) shown in Figure S1 was performed on paraffin embedded tissue sections following standard protocols. Endogenous peroxidase was blocked with dual endogenous enzyme block (DAKO, Glostrup, Denmark); tissues sections were incubated with primary antibody (α-SMA antibody, DAKO, Glostrup, Denmark) at 4° C overnight; sections were re-warmed at room temperature for 1 hour and washed with PBS before incubation with secondary goat anti-mouse antibody (DAKO, Glostrup, Denmark). Signal was visualized using 3,3’-diaminobenzidine (DAB) staining. 

### Reverse transcription quantitative real time PCR

Total RNA (775ng) isolated from C57BL⁄6 mouse liver was reversely transcribed using oligo dT primers (Roche cDNA synthesis kit, final volume: 60µl). Quantitative real-time PCR was performed on a LightCycler® 480 (Roche Applied Science) using 2µl cDNA, LightCycler® 480 SYBR Green I master mix (Roche) and respective PCR primers (Table 2). 

**Table 2:** Primers used for RT-qPCR.

Gene	**5’ Primer**	**3’ Primer**
Alb	GTCTTAGTGAGGTGGAGCATGACAC	GCAAGTCTCAGCAACAGGGATACAG
Arg1	GGAGGCCTATCTTACAGAGAAGGTC	CGAAGCAAGCCAAGGTTAAAGCCAC
Gys2	CCTCGATGGCTGTGATTTCTGACAC	CTTGGGCGTTATCTCTGTGCAGCAA
Gcgr	CACAGTGATCATGCAGTACGGCATC	CAAACAGACACTTGACCACCACCCA
Gapdh	CTTCAACAGCAACTCCCACTCTTCC	GGTTTCTTACTCCTTGGAGGCCATG
Gls2	CTTCTGCCAGAAGTTGGTGTCTCTC	CCGAGACATCTCCACTATATGCAGC
Gs	GCCAGGAGAAGAAGGGCTACTTTGA	GAGAGGGATCACTGGAAGTCTAGTC
G6pc	TCCTCCTCAGCCTATGTCTGCATTC	GAGAGAAGAATCCTGGGTCTCCTTG
Cyp2e1	CACCGTGTTCCGAGGATATGTCATC	ACACACGCGCTTTCCTGCAGAAAAC

qPCR was performed using the following protocol: 1 cycle pre-incubation: 5 min at 95°C, followed by 40 amplification cycles: 10s at 95°C, 10s at 60°C 20s at 72°C. For all samples melting curves were analyzed to ensure specificity of PCR products.

For statistical analysis, relative expression (RE) levels were calculated with the function (RE = 2-ΔΔCt), where ΔΔCt is the normalized difference in threshold cycle (Ct) number between the control sample and the CCl_4_- treated sample. Each Ct value was calculated from triplicate replicates. All samples were normalized to the expression of albumin. Relative expression levels were calculated from individual RE values of at least 2 independent experiments, and the standard error of the mean (SEM) was calculated from the standard deviation. Statistical significance was evaluated by Student’s t-test, comparing control samples to CCl_4_- treated samples.

## Results

Acute liver injury was induced by intraperitoneal injection of CCl_4_ in two groups of Balb⁄c mice, including 4 mice in each group. Mice from each group were sacrificed before, and 3h, 6h, 1d, 2d, 3d, 6d after injection, and livers were used to prepare paraffin embedded blocks and RNA extraction for quantitative real-time PCR.

Tissue sections from all time points were arranged on one slide to ensure equal conditions during *in situ* hybridization. Gene expression of two genes was visualized in one section using complementary digoxigenin and fluorescein labeled riboprobes, yellow staining for fluorescein labeled probes and violet staining for digoxigenin labeled probes. All *in situ* hybridization images were taken with a digital microscope at two magnifications to obtain an overview as well as detailed patterns of region specific gene expression. For optimal comparison, pictures of complementary areas from subsequent slices were recorded and complementary areas of liver tissue sections are shown for all hybridizations. 

**Figure 2:**
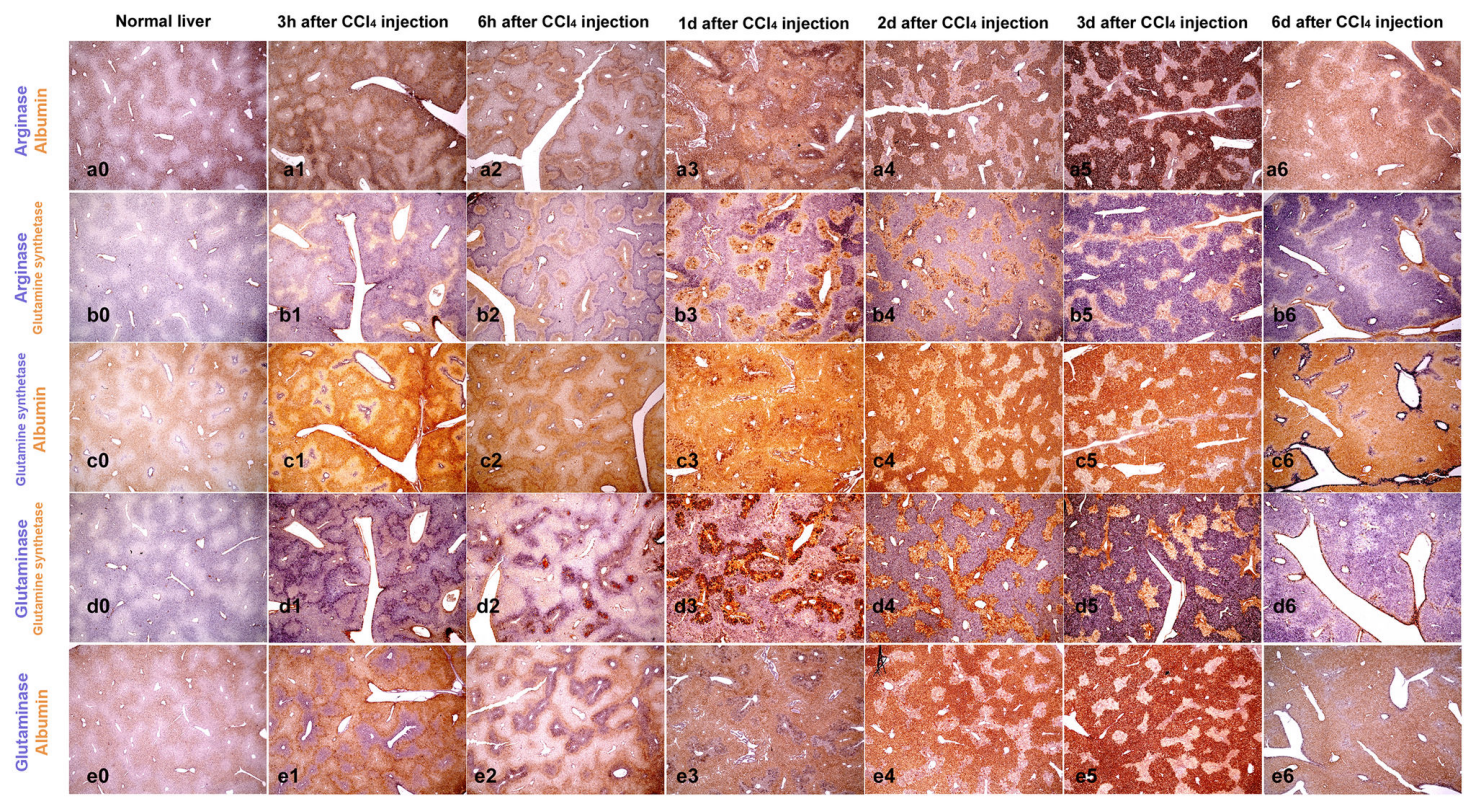
*In situ* hybridization for genes from nitrogen metabolism. *In* *situ* hybridization of mouse liver sections with probes for selected genes involved in nitrogen metabolism at different time points post CCl_4_ injection. In each panel, genes were visualized by dual staining with yellow and violet dye precipitation. Gene names are indicated on the left in the respective color. Co-staining for both genes in the same area resulted in dark “brown” staining. Pictures were captured with 4x objective.

**Figure 3:**
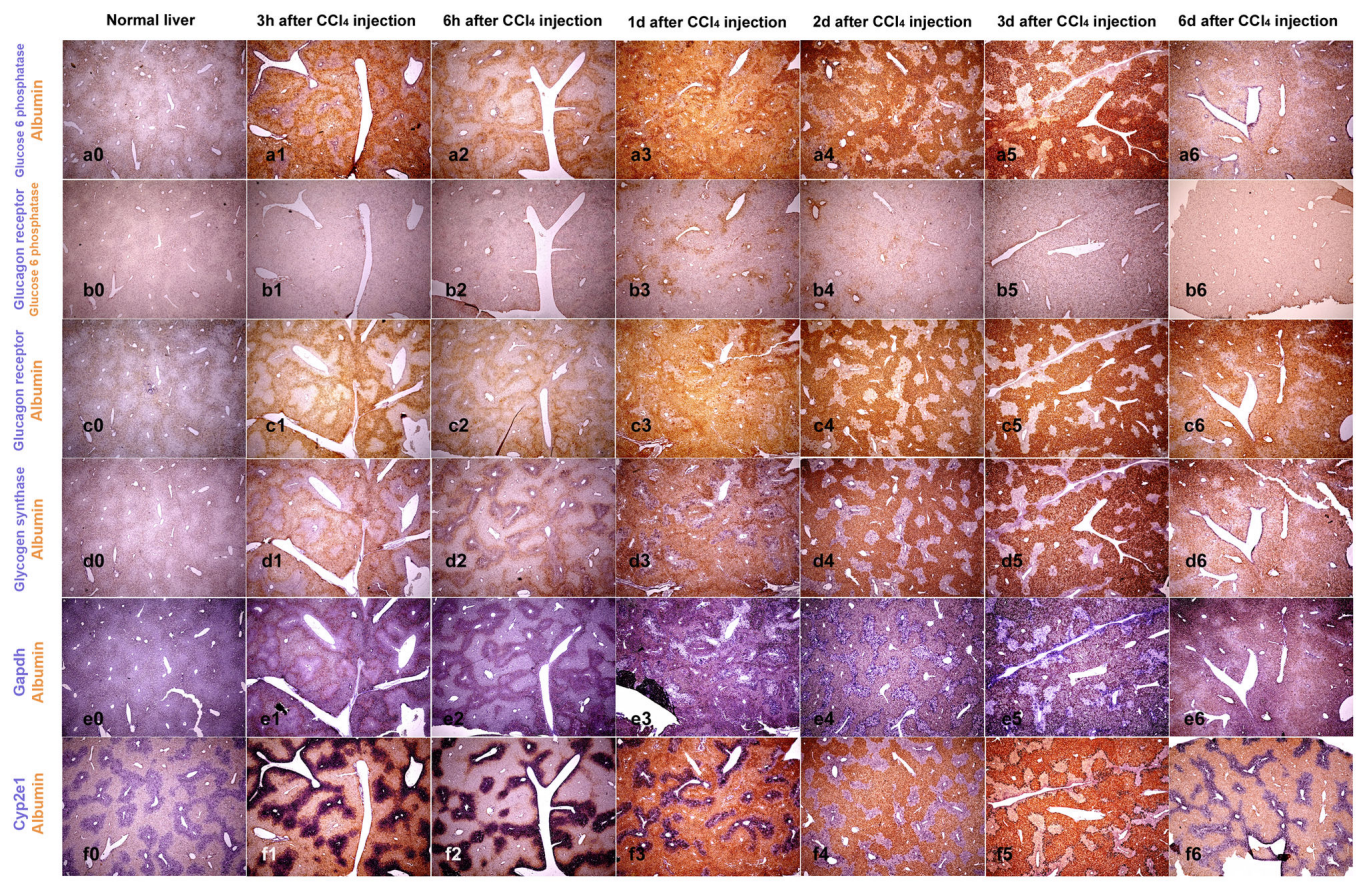
*In situ* hybridization for genes from carbohydrate metabolism. *In* *situ* hybridization of mouse liver sections with probes for selected genes of carbohydrate metabolism at different time points post CCl_4_ injection. Genes were visualized by dual staining with yellow and violet dye precipitation. Gene names are indicated on the left in the respective color. Co-staining for both genes in the same area resulted in dark “brown” staining. Pictures were captured with 4x objective.

**Figure 4:**
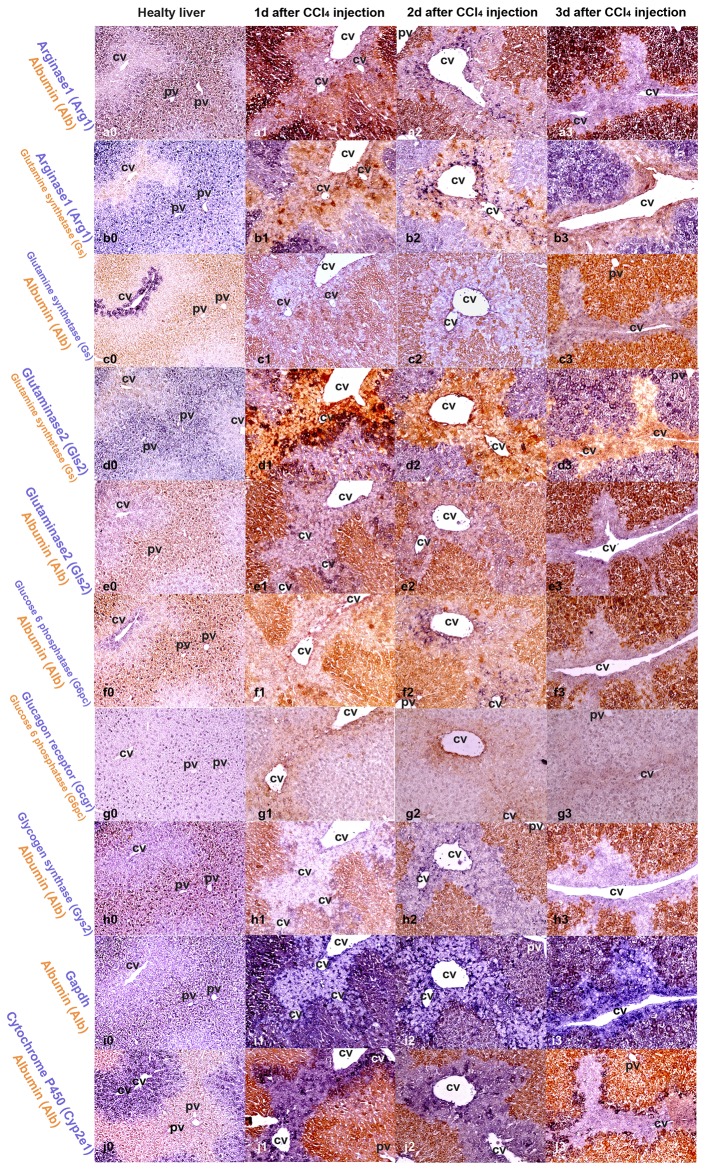
Higher resolution *in situ* hybridization images. *In* *situ* hybridization of mouse liver sections from untreated animals and form days 1 to 3 after CCl_4_ injection, with higher magnification (20x objective). Genes analyzed are indicated at the left in the respective color for each row. Co-staining for both genes in the same area resulted in dark “brown” staining. Specific areas of the liver tissue (acini) are marked: central vein (cv), portal vein/area (pv).

**Figure 5:**
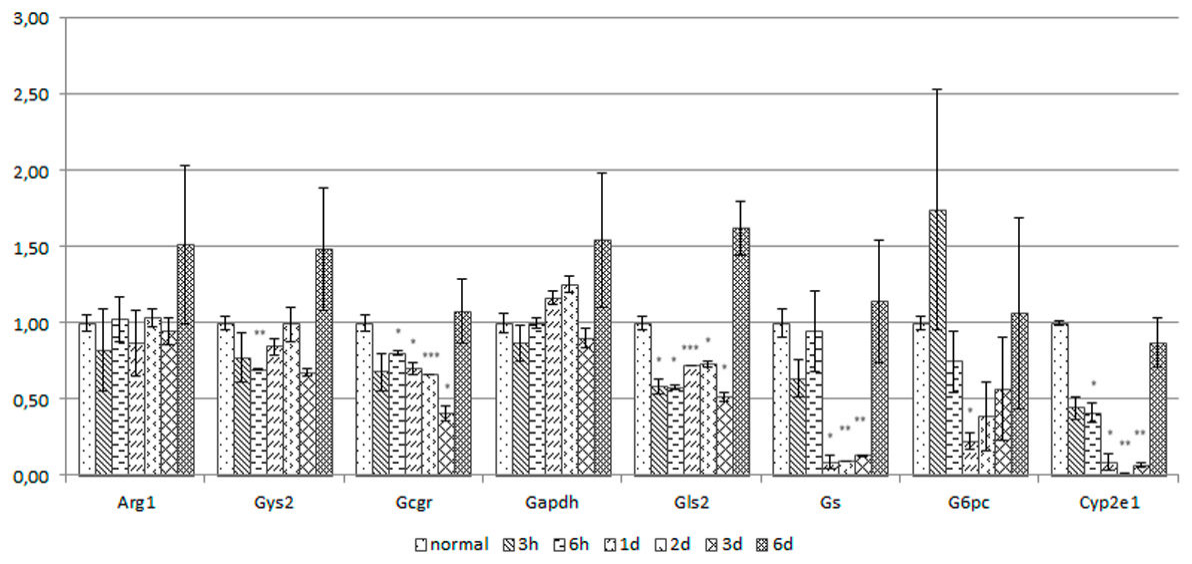
Quantitative analysis of over all gene expression levels. Analysis of total gene expression levels by reverse transcription quantitative real time PCR using primer pairs recognizing the following genes: arginase (Arg1), glycogen synthase (Gys2), glucagon receptor (Gcgr), Gapdh, glutaminase (Gls2), glutamine synthase (Gs), glucose-6-phosphatase (G6pc), cytochrom p450 2E1 (Cyp2e1). Albumin (Alb) was used for normalization.

ISH results are presented in figures 2, 3 and 4, showing gene expression of key enzymes of nitrogen metabolism and ammonia detoxification (Figure 2), glucose storage and release, and other genes involved in detoxification and basic cellular metabolism (Figure 3). *In situ* hybridization for albumin is included in all figures. In figure 4, higher magnification ISH pictures from days 1, 2 and 3 post CCl_4_ injection are presented to visualize area specific gene expression in more detail. Since ISH only provides relative gene expression values elucidating areas of high and low expression of respective genes, we also analyzed overall mRNA levels by RT-qPCR (Figure 5). 

In healthy liver, albumin is synthesized more in PPH than in PCH, which leads to a specific pattern of hepatic albumin expression visualizing the liver acini [25] (Figure 2, 3, and 4). In response to CCl_4_ treatment, albumin expression was even lower in PCH, visible in an increased signal difference between PPH and PCH for albumin mRNA. In contrast, at day 6 post CCl_4_ injection, albumin expression was more equally distributed than in untreated liver. 

In healthy liver, expression of enzymes involved in nitrogen metabolism and ammonia detoxification can be assigned to defined areas [9] (Figure 1A). *In situ* hybridizations for Arg1 and Gls2 show similar zonation in normal liver like albumin (Figure 2 a0, b0, d0 and e0), which fits well to the established functional association with PPH [26] (Figure 1A). Gs is expressed in PCH in 2 to 3 layers of hepatocytes around the pericentral vessel (Figure 2 c0), in good agreement with the published zonation of Gs activity [27, 8]. At early time points upon CCl_4_ treatment (3h and 6h) mRNA for Arg1 and Gls2 is detected throughout tissue sections, with very specific areas of higher expression, leading to a marked boundary between periportal and pericentral areas (Figure 2). At days 1 and 2 a more selective expression of Arg1 and Gls2 is observed, with sharp expression spots in the pericentral area. Strong signals for Arg1 in the pericentral area are clearly visible at day 2 in particular when analyzed with higher magnification (Figure 4 a2 and b2). Total mRNA levels of Arg1 analyzed by RT-qPCR were not changed relative to albumin until day 3 and increased only marginally at day 6 (Figure 5). In contrast, mRNA levels for Gls2 were immediately decreased and remained lower from 3h through day 3 after treatment and increased at day 6 (Figure 5). It should be noted, that Arg1 is not only expressed in hepatocytes and can be expressed also in macrophages [28]. The speckled signals observed at day 2 in the pericentral area (Figure 4 a2 and b2), therefore, could also stem from infiltrating macrophages during liver regeneration. Expression of Gs was also significantly changed during CCl_4_ treatment showing a strong expression around the pericentral vein at early time points (Figure 2 c0-2), which was lost at days 1, 2 and 3 (Figure 2 c3-5) and again clearly visible after 6 days (Figure 2 c6). Total Gs mRNA content in liver sections fits with these changes showing a significant decrease of Gs expression at days 1, 2 and 3 and a clear recovery at day 6 (Figure 5). 

Thus, while enzymes involved directly in the control of glutamine levels were decreased, the overall capacity to remove ammonia and generate urea may be retained throughout the toxic challenges.

Glucose storage and mobilization is another important role of hepatocytes in the liver, (Figure 1B). Glycogen synthase 2 (Gys2) is the enzyme for glycogen synthesis and glucose-6-phophatase (G6pc) is needed to release glucose. On total mRNA level, expression of Gys2 is only marginally changed and shows a similar pattern of expression like Arg1 (Figure 5). G6pc in contrast was induced after 3h, significantly reduced at day 1 and followed by a clear recovery at late time points (Figure 5). Glucagon receptor (Gcgr) involved in the regulation of both enzymes appears to be down regulated until day 3 and recovered as well at day 6 (Figure 5). 

Looking at the spatial distribution in liver tissue, a quite uniform expression of the Gcgr is observed (Figure 3 c), also clearly visible at higher magnification (Figure 4 g). The strong signal of albumin obscures this homogenous signal, which is however clearly visible in co-staining with G6pc (Figure 3 b). Like Gcgr, Gys2 mRNA is found equally distributed in all areas of the tissue, resulting in virtually identical hybridization pictures when co-stained with albumin (Figure 3 d0, and 4 h). Looking at the spatial expression of G6pc very distinct expression pattern are visible at days 1, 2 and 3 (Figure 3 b3-5). At higher resolution (Figure 4 f1-3 and g1-3) G6pc expression in the “damaged” pericentral area is clearly visible. It should be noted that the probe used for hybridization is specific for G6pc1, the isoform expressed by hepatocytes in the liver, and should not detect other glucose-6-phosphatase isoforms. 

Gapdh is required for utilization of glucose for energy production. Because of this basic function, it is considered a housekeeping gene and used as gene expression reference. In fact total mRNA levels were not significantly changed during treatment and only a slightly higher expression relative to albumin was detected at day 6 (Figure 5), similar to expression of Arg1 and Gys2. Due to its central role in energy generation, Gapdh should identify cells with high energy requirements in the tissue. In liver section of untreated mice, Gapdh is homogeneously expressed in pericentral and periportal areas. Upon CCl_4_ treatment distribution of Gapdh mRNA fits to the pattern of albumin in the first 6h. At later time points, days 1 to 3, expression of Gapdh was again elevated in the damaged areas (Figure 3 e3-5, and 4 i0-3) clearly reflecting continuous Gapdh expression. Interestingly, some cells express Gapdh with high intensity (Figure 3 e3-5, and 4 i1-3). This staining clearly demonstrates that metabolically active cells are present within the damaged area. 

Taken together, the following picture emerges. While expression of Gys2 is not much changed and quite uniform in the tissue, expression of Gcgr is uniformly reduced, G6pc shows the most dynamic pattern, after an immediate induction at 3h, expression is significantly reduced at day 1 which is followed by a continuous increase until day 6. Interestingly, ISH clearly shows that expression of G6pc is not uniformly lost. While over all expression is reduced (Figure 5), a high level of expression is retained in the damaged area (Figure 3, 4, and Suppl.-Figure S1). The association with a general high metabolic activity is further reflected by expression of Gapdh in the damaged area (Figure 3, and 4). 

The specific gene expression response to the toxic challenge becomes even clearer looking at expression of Cyp2e1 required for CCl_4_ metabolism. Overall expression of Cyp2e1 is down regulated immediately after CCl_4_ exposure with lowest levels at days 1 to 3 followed by a recovery at day 6. Looking at area specific expression by ISH clear expression patterns can be seen showing a local increase of Cyp2e1 expression in the pericentral area (Figure 3 f). At higher magnification (Figure 4 j) strong signals for Cyp2e1 mRNA are clearly visible in specific areas, which define a boundary along the area with higher albumin expression. 

## Discussion

It is widely accepted that treatment with an acute toxic dose of CCl_4_ leads to massive necrotic cell death in the pericentral area and that regeneration involves degradation and removal of the remaining cell debris and repopulation of the necrotic area by proliferating hepatocytes from adjacent unaffected areas [2,29,30,31,32]. This assumption is based on the observation that CCl_4_ treatment leads to a strong increase of liver enzyme serum levels and on histological analysis of liver tissue in response to CCl_4_ treatment showing changed morphology, reduced liver enzyme activity in the damaged pericentral area [2,33], and visualization of apoptotic cell nuclei and transient caspase activation [34].

**Figure 6:**
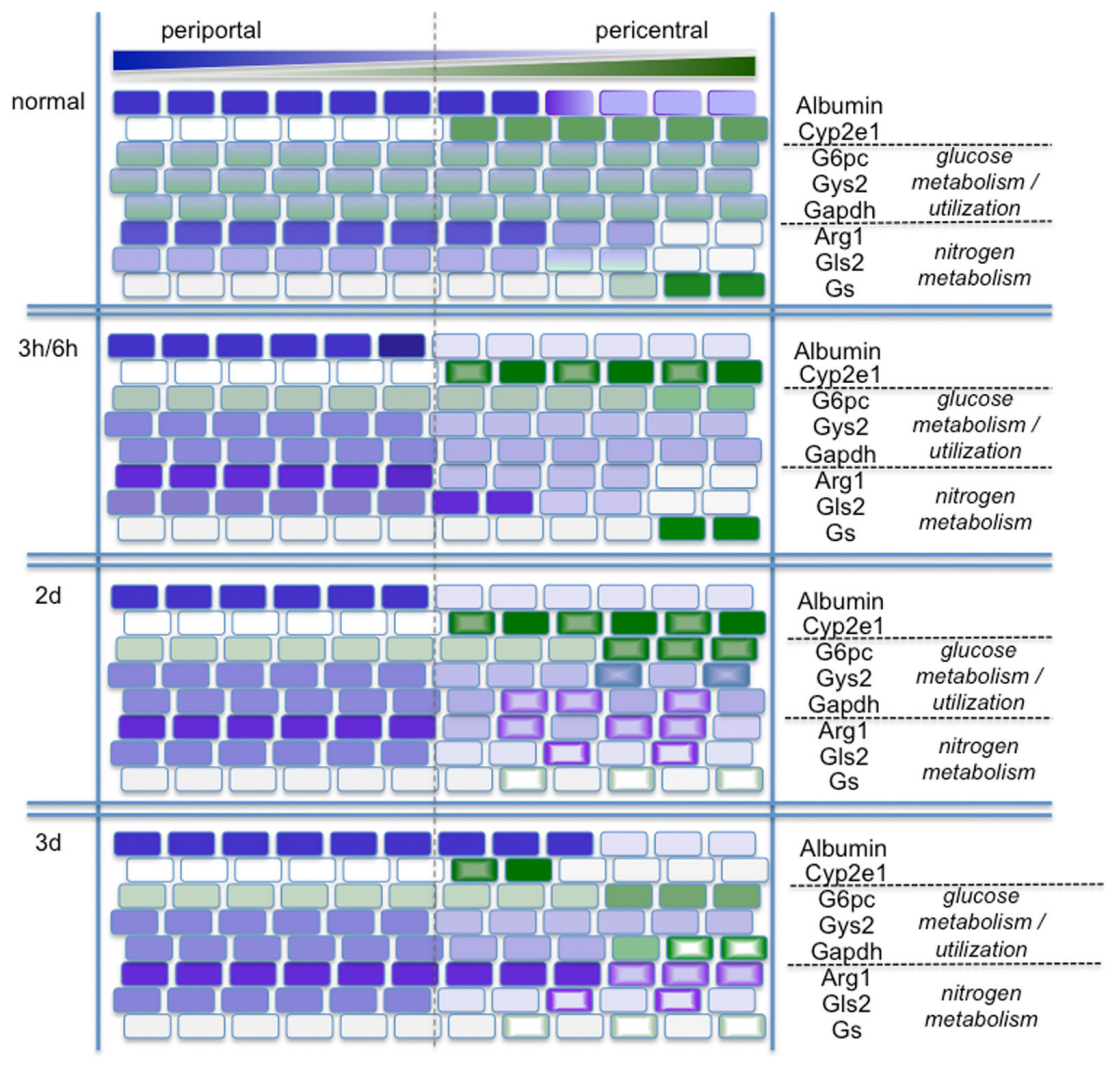
Graphical summary of the area specific gene expression patterns. Results from the ISH analysis are summarized into 4 conditions. Untreated liver (normal), and after 3h and 6h, 2 days (2d) and 3 days (3d) upon CCl_4_ treatment. Intensity of the color reflects the relative intensity of gene expression for each gene (intense/dark color: strong/high signal). The intensities only visualize the relative distribution of the respective mRNA along the axis from the periportal area to the pericentral vein (blue: higher periportal: green; higher pericentral). Open cells (circular light to dark coloring) are used to indicate non homogenous speckled patterns observed in the ISH images.

In this study, we analyzed expression of enzymes involved in nitrogen metabolism and glucose storage and release by *in situ* hybridization in livers of CCl_4_ treated mice, a well established model for liver cell damage [35], and observed area specific adjusted gene expression, summarized in Figure 6. Our results clearly show continuous gene expression, which requires de novo synthesis of mRNA, in the area around the pericentral vein throughout the time course analyzed. De novo synthesis of mRNA can only occur in viable cells and reflects gene expression in the specific area. (Correlation of mRNA and protein signal during CCl_4_ treatment is exemplary shown for alpha-smooth muscle actin (aSma) in Figure S1 A). The time dependent changes in gene expression patterns observed, indicate that larger numbers of cells survived in the pericentral area and contributed to repair and regeneration. Nevertheless, CCl_4_ treatment led to visible tissue damage with loss of cell-cell interactions, and infiltration of blood cells into the damaged area around the pericentral vein (data not shown). Combining our observation with previously published results showing cell death around the central vein after CCl_4_ treatment [34|, we assume, that a more complex response occurs in the damaged area including cell survival and a specific cellular response. A clear indicator that liver parenchymal cells retained vital functions is the expression of the hepatocyte specific isoform of glucose-6-phosphatase (G6pc) in the damaged pericentral area (Figure 3, 4, and Suppl.-Figure S1), despite a strong reduction of total G6pc mRNA levels analyzed by RT-qPCR (Figure 5), It is important to note, that this area specific expression of G6pc is complemented by increased Gapdh mRNA signals in the same area albeit not necessarily in the same cells. 

Selective expression was also observed for genes required to eliminate ammonia and maintain glutamine levels. While overall expression of Arg1 is not significantly changed, it is increased in the damaged area at later time points. The reorganization of Arg1 expression not only shows a time scale of late recovery but also a very specific pattern of tissue distribution during the time course analyzed. While zonation of arginase expression at early and late time points overlaps with albumin, indicating hepatocyte specific expression, a quite different picture is observed at day 2, where Arg1 is expressed in a speckled pattern in the damaged area. Since Arg1 can be expressed in many cell types, this signal could not only come from hepatocytes, but also from other cells, including stellate cells or infiltrating blood monocytes. It should be noted that glutamine synthase is also expressed in a speckled pattern at days 1 and 2. These speckled signals do not overlap, indicating that arginase and glutamine synthase are expressed in different cell populations in the damaged area.

The most compelling adjustment of gene expression upon CCl_4_ treatment is the local adjustment of Cyp2e1 expression over time. Cyp2e1 was expressed throughout the liver but expression was higher around the pericentral vein in untreated animals and after recovery at day 6. Immediately after treatment, at early time points, Cyp2e1 expression was locally increased in the pericentral area, although overall expression in total liver lysates was reduced. At later time points, zonation of Cyp2e1 expression was further changed and a sharp and clear boundary like expression pattern emerged, separating the albumin producing periportal area and the damaged pericentral region (Figure 4, 6, and Suppl.-Figure S1). Although activation of CCl_4_ by Cyp2e1 contributes to the observed severe damage, the very specific pattern of Cyp2e1 expression could ensure efficient transformation and detoxification of CCl_4_ in the pericentral area to prevent further spread of the toxic agent into other areas, thereby limiting overall tissue damage supporting regeneration. 

## Conclusion

Following gene expression required for nitrogen metabolism and glucose utilization upon CCl_4_ damage in the liver, we observed a rapid adjustment of expression patterns on both, the overall level and the spatial distribution. Our findings confirm, that CCl_4_ mediated damage occurs around the central vein. Surprisingly, CCl_4_ damage does not immediately result in severe cell death but rather leads to a rapid adjustment of expression patterns to the toxic challenge, which we assume limits toxicity and supports rapid recovery of the liver tissue. This response includes, among others (1), strong local expression of the detoxifying enzyme Cyp2e1 presumable to limit further distribution of CCl_4_ in the tissue (2), increased turnover of nitrogen (3), local mobilization of glucose from hepatocytes in the damaged area, and (4) high levels of glucose utilization, reflecting intense metabolism required for repair and recovery in the damaged area. Our results do not exclude cell death of severely damaged hepatocytes and formation of new hepatic tissue during recovery. Nevertheless, liver cells remain viable in the damaged area and adjust gene expression to orchestrate protection from further damage and to enable efficient recovery in the damaged area, which reaches normal patterns of gene expression, e.g. albumin production, at day 6 after CCl_4_ treatment.. 

Thus, zonation of biochemical functions in the liver not only plays an important role for basic liver functions, but is also needed to cope with damage. This could limit tissue damage to smaller areas. In addition, sustained viability and adjusted gene expression patterns of all liver cell types could contribute to a more efficient regeneration of the damaged region.

## Supporting Information

Figure S1 **Comparison of *insitu* hybridization and immunohistochemistry.** (A) *In* *situ* hybridization for α-smooth muscle actin (aSma) and albumin in comparison with detection of aSma by immunohistochemistry and Eosin staining on consecutive liver sections. (B) Higher resolution images of *in* *situ* hybridization for Cyp2e1, albumin and G6pc (single staining) together with DAPI staining of consecutive liver sections. Specific areas are marked: central vein (cv), portal vein/area (pv).
(TIF)
