# Supplementary figures and images for: Zonation of Nitrogen and Glucose Metabolism Gene Expression upon Acute Liver Damage in Mouse
Source: PLoS One. 2013 Oct 17;8(10):e78262. doi: 10.1371/journal.pone.0078262 (PMC3798318; doi:10.1371/journal.pone.0078262)

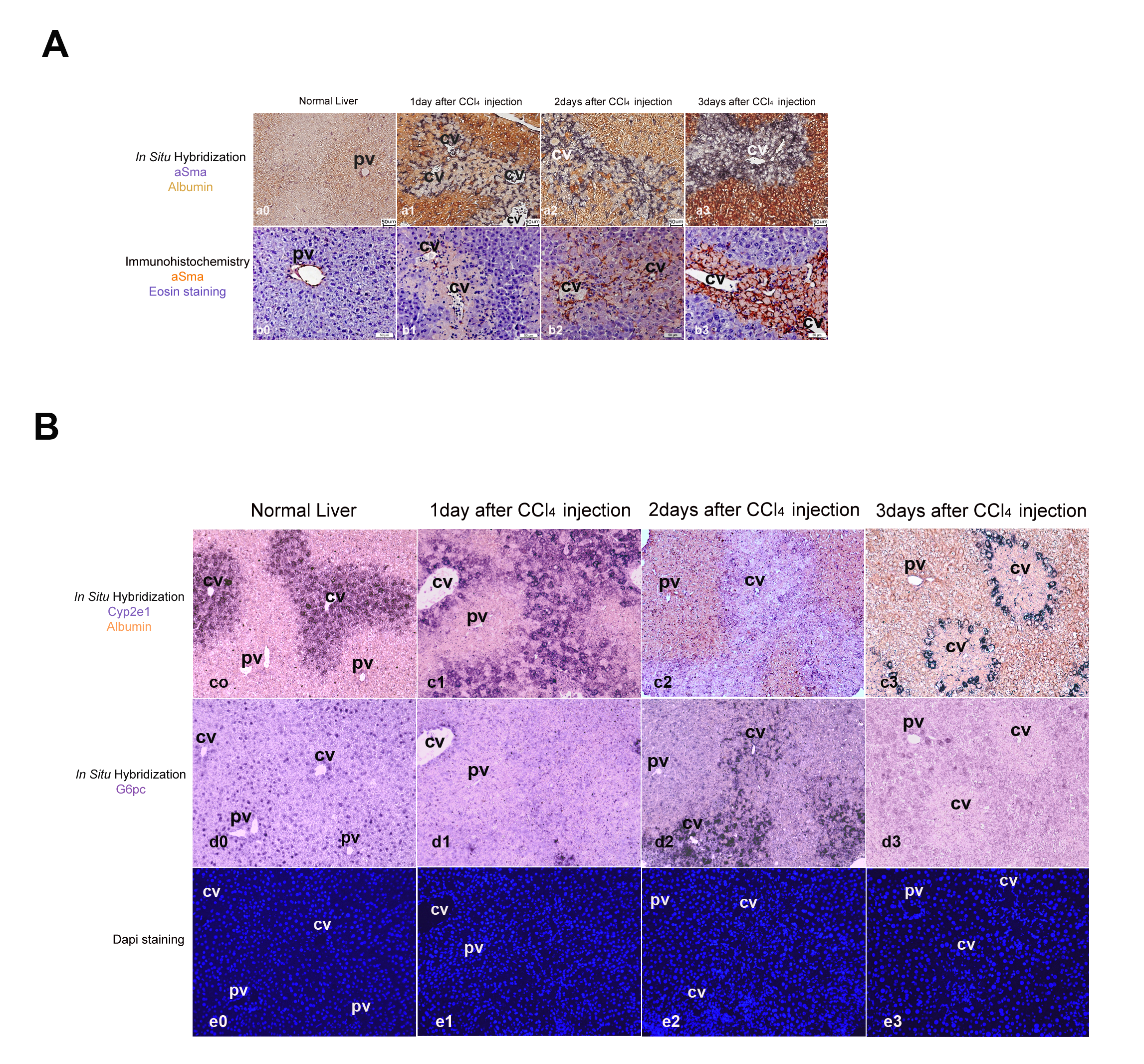

Supplement: Figure S1 — Comparison of insitu hybridization and immunohistochemistry. (A) In situ hybridization for α-smooth muscle actin (aSma) and albumin in comparison with detection of aSma by immunohistochemistry and Eosin staining on consecutive liver sections. (B) Higher resolution images of in situ hybridization for Cyp2e1, albumin and G6pc (single staining) together with DAPI staining of consecutive liver sections. Specific areas are marked: central vein (cv), portal vein/area (pv). (TIF) [file pone.0078262.s001.tif]
